# Do rational numbers play a role in selection for stochasticity?

**DOI:** 10.3389/fncom.2014.00113

**Published:** 2014-09-25

**Authors:** Robert Sinclair

**Affiliations:** Mathematical Biology Unit, Okinawa Institute of Science and TechnologyOnna-son, Okinawa, Japan

**Keywords:** stochastic development, rational numbers, cell fate determination, tissue patterning, retina

## Abstract

When a given tissue must, to be able to perform its various functions, consist of different cell types, each fairly evenly distributed and with specific probabilities, then there are at least two quite different developmental mechanisms which might achieve the desired result. Let us begin with the case of two cell types, and first imagine that the proportion of numbers of cells of these types should be 1:3. Clearly, a regular structure composed of repeating units of four cells, three of which are of the dominant type, will easily satisfy the requirements, and a deterministic mechanism may lend itself to the task. What if, however, the proportion should be 10:33? The same simple, deterministic approach would now require a structure of repeating units of 43 cells, and this certainly seems to require a far more complex and potentially prohibitive deterministic developmental program. Stochastic development, replacing regular units with random distributions of given densities, might not be evolutionarily competitive in comparison with the deterministic program when the proportions should be 1:3, but it has the property that, whatever developmental mechanism underlies it, its complexity does not need to depend very much upon target cell densities at all. We are immediately led to speculate that proportions which correspond to fractions with large denominators (such as the 33 of 10/33) may be more easily achieved by stochastic developmental programs than by deterministic ones, and this is the core of our thesis: that stochastic development may tend to occur more often in cases involving rational numbers with large denominators. To be imprecise: that simple rationality and determinism belong together, as do irrationality and randomness.

## Introduction

Aristotle tells us that the Pythagoreans believed that the principles of mathematics were the principles of all things (Primavesi, 2012). Of special importance to them were the rational numbers, which were associated with harmony in music.

I wish to propose that these same rational numbers may help us to understand conditions under which a developmental process may tend to appear to be random from some point of view, where I use the word “random” in the sense of something which is not readily predicted. Wu et al. (2013) provide a directly relevant example of a synthetically engineered mechanism for stochastic differentiation, which is an example of what I mean by “stochastic” (and “random”): the critical point is that the mechanism effectively amplifies what one might call environmental noise, and makes a decision based upon that amplified signal. I will also assume that natural selection can distinguish between deterministic and stochastic development, and, in the case of stochastic development, can select or tune for particular probabilities.

Before going into the details of the hypothesis, is there any reason to think that one can infer anything useful at all about living organisms on the basis of what may appear to be a type of numerology? There are two historical cases which illustrate the potential of such an approach, one from chemistry and one from palaeontology.

John Dalton is famous for, among other things, his law of multiple proportions, which is now a part of any basic chemistry course, but was advanced theory 200 years ago, before atomic theory was generally accepted and well before the structures of molecules were known. In a standard modern textbook (Brown et al., 2009), it is formulated as “If two elements A and B combine to form more than one compound, the masses of B that can combine with a given mass of A are in the ratio of small whole numbers”. Did Dalton himself think in terms of simple rational numbers? In Chapter IV of Part II of Dalton’s book introducing a new system of chemical philosophy (Dalton, 1810), we find “Thus the law of chemical synthesis is observed to be simple, and always limited to small numbers of the more simple principles forming the more compound”. In the appendix of the same work, he writes, in a reference to work by Gay Lussac, more clearly of “an hypothesis that all elastic fluids combine in equal measures, or in measures that have some simple relation one to another, as 1 to 2, 1 to 3, 2 to 3, & c.” and, to illustrate that he was very much aware that he was making inferences about something as complex as chemical synthesis but using simple rational numbers, “In no case, perhaps, is there a nearer approach to mathematical exactness, than in that of 1 measure of oxygen to 2 of hydrogen; but here, the most exact experiments I have ever made, gave 1.97 hydrogen to 1 oxygen”. No-one would deny the complexity of chemical reactions, nor would anyone deny the predictive power of Dalton’s law of multiple proportions. This is one historical precedent for the use of simple rational numbers as a step towards deeper understanding of complex processes.

As my second historical example, I wish to use the case of the “conodont animal” (Sweet and Donoghue, 2001), which remained mysterious until quite recently, for the simple fact that the feeding elements or “teeth”, which are usually the only parts preserved, are only rarely found in the relative positions they would have occupied in the living animal (but see Briggs et al., 1983; Goudemand et al., 2011 for some beautiful exceptions). There was a time when these elements were individually classified, as if each one represented a new species, but it gradually became clear that one should treat assemblages of these elements as single units (i.e., as the partial remains of single animals). A crucial link in the argument for assemblages was the fact that large collections of the elements tended to show simple proportions between the numbers found of the different types. For example, Du Bois (1943) stated that “The standard conodont complement of these structures apparently includes a pair of polygnathids, a pair of bryantodids, and at least four, possibly more, pairs of hindeodellids. The hypothetical proportion of 1:1:4, indicated by a study of the assemblages is borne out by a study of the isolated individuals. Of 479 separate teeth, 67 have been classified as *Ozarkodina*, 108 as *Streptognathodus*, and 304 as *Hindeodella*”. The fact that the ratios may not appear to us to have matched very well does not overshadow the importance that such ratios had in justifying the hypothesis that assemblies of elements, rather than the elements themselves, could correspond to the animal. Once again, we see that simple proportions can have something useful to say about something as complex as the anatomy of an unknown animal. To give this example the appropriate perspective, note that Briggs et al. (1983) wrote “The nature of the conodont animal has perhaps been the subject of more speculation than any other question in palaeobiology”.

I do understand that even these examples will still fail to convince some readers that hypotheses of the type I am putting forward can actually be of use, but I will refrain from listing any more (although Mendelian genetics comes to mind). I am aware of the attractiveness of particularism as opposed to broad theory (Gremillon et al., 2014), but I am personally convinced that theory can be powerful, even if it appears to ignore important facts. Charles Darwin’s original statement of his Theory of Natural Selection continues to be *useful*, although it never included the vast majority of the now known facts of molecular biology. The difference between a hypothesis and a collection of raw data is often the selective focus of the hypothesis, which emphasizes some facts while appearing to ignore others. This should not be confused with true ignorance. To be specific, the hypothesis I present here is not formulated in terms of energy or structure. After much consideration, I made a conscious decision to focus on proportions instead.

The intention of this work is to suggest a novel framework for thinking about the relationship between natural selection and stochasticity in development. I hope that it will be useful in designing and interpreting experiments. I have no intention of falsely elevating my hypothesis to anything approaching a law. There are good reasons to expect exceptions, and, to be specific, competence for DNA uptake in bacteria may be one (see Johnston and Desplan, 2010 for a description and references). Instead, I hope my hypothesis will be useful in the same sense that the thought “If I drop it, it will fall” is useful, even though the helium balloons at some children’s parties openly contradict it. In what follows, I will provide a number of examples, which are intended to illustrate how I suggest that the hypothesis should be applied, and where I think it may apply.

The Drosophila compound eye provides excellent examples of both deterministic assembly and stochastic development, in which we can clearly see the proposed relationship of developmental mechanism with rational numbers between 0 and 1.

A normal Drosophila ommatidium contains exactly eight photoreceptor neurons. Only photoreceptor neurons R1 to R6 express the major Drosophila rhodopsin Rh1. Therefore, the fraction of photoreceptor neurons in a Drosophila compound eye which express Rh1 is a rational number: 6/8 = 3/4 = 0.75 = 75%. If we include the fact that there is a single R8 founder cell, being one out of eight, we find the lowest common denominator is 8. The simplicity of ommatidial composition is reflected in the simplicity of the rational numbers 3/4 and 1/8 with their small lowest common denominator (i.e., 8). Assembly essentially always follows the same pattern within an ommatidium. On the other hand, R7 photoreceptors express either Rh3 or Rh4 rhodopsin, but here the fraction is not easily written down as a rational number, instead being a value in the range of 60% (Bell et al., 2007) to 70% (Wernet et al., 2006) for Rh4. How can this be achieved? Since 70% = 7/10, one might imagine coordinated groups of 10 ommatidia, a specific set of seven of which have R7 neurons expressing Rh4, but this implies tightly coordinated groups of 80 (7/10 = 56/80, where 80 = 8 × 10, since we are talking about 10 ommatidia, but these already contain eight cells each) photoreceptor neurons, something likely to be prohibitive from a developmental point of view. There is less numerical simplicity, as reflected in the much larger denominator (8 × 10). This is true even if we take 60% = 3/5 as the relevant fraction, since it would still imply coordination of groups of 8 × 5 = 40 cells.

However, a stochastic mechanism can be tuned (Wu et al., 2013 provide an idea of how) to a fraction of around 60% or 70%, and the complexity of the mechanism is unlikely to differ greatly as a function of the exact target figure. Since we know that the choice between Rh3 and Rh4 is indeed stochastic, it would appear that the cost/complexity of a stochastic mechanism may somehow be lower than that of a deterministic mechanism coordinating 80 cells. I suggest that, in evolutionary terms, there may be selective pressure to reduce unnecessary developmental complexity, and this may be related to the fact that the observed choice here is a stochastic one.

The hypothesis is, that *if the fractions of cell types within a specified tissue are best approximated by rational numbers with a large lowest common denominator, then development may tend to be stochastic rather than deterministic*, and, that *this tendency should become more pronounced as the lowest common denominator increases*.

## “Simple” rational numbers

Moving away from the compound eye of Drosphila, let us return to the theoretically more basic problem of tissue consisting of different types of cells which are not strictly arranged in units (such as an ommatidium). Here, the relationship between properties of the “simple” rational numbers and the proposed tendency towards stochastic development can be made more directly. The fact that rational numbers with bounded denominators are distributed unevenly in any large interval is useful in understanding the hypothesis, and is illustrated in Table 1.

**Table 1:** **Gaps between “simple” rational numbers**.

Simple Fraction	Percentage	Difference from previous
1	100.0%	
9/10	90.0%	100.0–90.0 = 10.0%
8/9	88.9%	90.0–88.9 = 1.1%
7/8	87.5%	88.9–87.5 = 1.4%
6/7	85.7%	87.5–85.7 = 1.8%
5/6	83.3%	85.7–83.3 = 2.4%
4/5 = 8/10	80.0%	83.3–80.0 = 3.3%
7/9	77.8%	80.0–77.8 = 2.2%
3/4 = 6/8	75.0%	77.8–75.0 = 2.8%
5/7	71.4%	75.0–71.4 = 3.6%
7/10	70.0%	71.4–70.0 = 1.4%
2/3 = 4/6	66.7%	70.0–66.7 = 3.3%
5/8	62.5%	66.7–62.5 = 4.2%
3/5 = 6/10	60.0%	62.5–60.0 = 2.5%
4/7	57.1%	60.0–57.1 = 2.9%
5/9	55.6%	57.1–55.6 = 1.5%
1/2 = 2/4 = 4/8 = 5/10	50.0%	55.6–50.0 = 5.6%

*Rational numbers with denominators up to 10 are shown in descending order, along with the sizes of the gaps between consecutive numbers. Due to symmetry, only fractions between 1/2 and 1 (inclusive) are shown*.

The gaps in Table 1 indicate intervals in which “simple” rational numbers cannot be found. 64% is in one of the larger gaps, for example. This suggests that tissues requiring such a fraction may not lend themselves to growth in a straightforward deterministic manner. While these gaps do provide a useful image of how the hypothesis is to be applied, the more precise definition of the hypothesis remains purely in terms of the sizes of denominators of rational numbers.

### Retinal cell lineages

The retina is a relatively easily accessible brain tissue belonging to the vertebrate central nervous system. It is composed of a number of different cell types, and the mechanism by which the appropriate proportions of retinal neurons are derived from retinal precursor cells has been studied in a number of different vertebrate model organisms (see the review by Johnston and Desplan, 2010). For example, retinal lineage tracing in zebrafish by He et al. (2012) has shown that the mechanism is stochastic.

The experiments and analysis of Gomes et al. (2011) provide us with a very clear case study, due not least to the large number of careful experiments they performed over a period of more than 2 years. Their paper contains a great amount of detail, which we can make use of here. In particular, they observed four different retinal cell types being generated by a non-deterministic process from the retinal progenitor cells. These four differentiated cell types had frequencies of 341/462 = 73.8%, 59/462 = 12.8%, 49/462 = 10.6% and 13/462 = 2.8%. From Table 1, it is clear that several of these fractions fall within the larger gaps. Alternatively, the lowest common denominator (462) is very large, which, according to our hypothesis, should translate into a tendency towards a stochastic mechanism. Gomes et al. (2011) were able to conclude that the mode of cell division for retinal progenitor cells is stochastic, and that cell fate specification is based mainly on stochastic choices, but the simplest stochastic model was not consistent with all of their data. Despite the enormous effort put into their experiments, the rejection of the simplest stochastic model was on the basis of only very small (but statistically significant) excesses in the numbers of observed triplets of cells. Although it is perhaps unrealistic to hope for, it would be very useful to have even further experiments performed at some point in the future, to be sure that their final conclusion was unaffected by small sample errors (they were forced to exclude video recordings in which cells were not visible, became invisible or could not be resolved etc., and any possible biases, however small, resulting from this are difficult to estimate).

## Generality of the hypothesis

### Rare cell or organism types

The largest gap in Table 1 is for fractions near unity. By symmetry, this implies that an equally large gap appears near 0. Our hypothesis predicts that fractions near (but not equal to) 0 should tend to be associated with stochastic mechanisms.

The hypothesis is, by its nature, not restricted to neurons. To test whether it might apply to other contexts, it is interesting to examine some examples from other areas of biology.

It is known that an extremely small fraction of wild-type bacterial cells will be found to be in a dormant state, and that these cells are responsible for resistance to antibiotic treatment, since their reduced metabolism allows them to survive. Johnston and Desplan (2010) and Maisonneuve et al. (2013) provide more detailed descriptions of this phenomenon of persistence. The fractions involved are in the range 10^−6^ to 10^−4^. Our hypothesis would therefore predict a stochastic mechanism, rather than the coordination of up to millions of cells. This is in fact what is observed. Wild-type cells switch stochastically to a dormant state and also revert to their normal proliferating state after some time. The switching probability is at an appropriate low level to keep the fraction of cells in the dormant state at the very small target values.

This is to be contrasted with the case of heterocyst formation, a response to nitrogen starvation, along cyanobacterial filaments. Heterocysts are differentiated cells which differ from all the other cells in the filament, and are found approximately in the proportion 1:10 (Yoon and Golden, 1998). The heterocysts are distributed remarkably evenly along the filaments. Our hypothesis would predict that this would be a case where a deterministic mechanism may underlie cell differentiation, since the denominator (11) of the associated rational number (1/11) is still reasonably small (similar to the number (8) of cells in the Drosophila ommatidium). What is actually observed (see Figure 3D of Yoon and Golden, 1998), is a distribution of numbers of vegetative cells between heterocysts which peaks at 10, but is broad. Nonetheless, the distribution is not consistent with a random model of cell-fate determination, for which one would expect to see an approximately exponential distribution with frequent closely spaced heterocyst pairs (as is observed in some mutant strains, as illustrated by Figure 3E of Yoon and Golden, 1998).

A further example is provided by the worm *C.elegans*. Approximately one in 300 is a male, and, once again, our hypothesis predicts a stochastic mechanism for the corresponding large denominator. Indeed, males are the product of rare, “accidental” loss of an X chromosome (Hodgkin, 2002).

### Human sex ratios

It is commonly believed that the human ratio of male to female live births is 1:1. If that were the case, our hypothesis would predict a deterministic mechanism for sex determination, presumably in terms of pairs. In other words, if the sex ratio were under very strong selection to be identically 1, then one might predict that humans would almost always bear twins, one male and one female. Clearly, this is not the case!

Interestingly, the human sex ratio is in fact stably greater than 1, usually around 1.05 (Mathews and Hamilton, 2005). In other words, for every 200 live births, one can expect 105 males and 95 females. The fraction 105/200 = 21/40 appears in the gap near one half in Table 1, and our hypothesis predicts a stochastic mechanism, which is of course a source of much entertainment for humans.

## Closing

From an evolutionary perspective, it is clear that so potentially dramatic a change from a deterministic to a stochastic developmental mechanism, or the reverse, should be a rare event. This implies that there is no reason to expect that all tissues in all organisms, to which this hypothesis could apply, must automatically be assumed to be using stochastic developmental mechanisms according to a simple numerical assay of cell type abundances. Also, there are many possible selective forces which can and do influence the choice between determinism and stochasticity, and alternative ways to interpret this choice, a recent example of which is provided by Fisek and Wilson (2014) and Friedrich et al. (2014). The possibility of migration must also always be considered. Instead, what is being suggested here is that there may be a tendency, which is likely to manifest itself most clearly in a survey of many tissue types in many organisms. This tendency is, according to the hypothesis presented here, likely to reflect the structure of Table 1, in that cell type frequencies in gaps between “simple” rational numbers would be expected to be associated with stochastic development, statistically speaking.

I would like to finish with a question: Should we expect programmed cell death under stochastic control (Spencer et al., 2009) to be selected for in nervous system development?

## Conflict of interest statement

The author declares that the research was conducted in the absence of any commercial or financial relationships that could be construed as a potential conflict of interest.
